# An Integrated DNA Nanoprobe for Intranuclear Imaging and in Situ Profiling of OGG1 Activity

**DOI:** 10.1002/advs.75847

**Published:** 2026-05-27

**Authors:** Mingzhu Zhao, Xuemei Sun, He Li, Bangming Wang, Mengting Pan, Zhen Ma, Rong‐Mei Kong, Weiheng Kong, Yan Zhao, Fengli Qu

**Affiliations:** ^1^ Key Laboratory of Life‐Organic Analysis of Shandong Province School of Chemistry and Chemical Engineering Qufu Normal University Qufu Shandong P. R. China; ^2^ School of Molecular Medicine Hangzhou Institute for Advanced Study University of Chinese Academy of Sciences Hangzhou Zhejiang P. R. China; ^3^ Department of Intensive Care Unit Jining No. 1 People's Hospital Jining Shandong P. R. China

**Keywords:** 8‐Oxoguanine, nuclear targeting, OGG1 activity, oxidative DNA damage, signal amplification

## Abstract

Accurate quantification of 8‐oxoguanine DNA glycosylase‐1 (OGG1) activity in the cell nucleus is crucial for evaluating the DNA oxidative damage marker 8‐oxoguanine (8‐oxoG). However, due to the difficulty in nuclear localization and the predicament of strong signal output, subcellular imaging has not yet been achieved. Herein, we report a nuclear‐targeted DNA triangular prism nanoprobe (TP‐SA) designed to overcome the aforementioned limitations via a dual‐pronged strategy. TP‐SA integrated an AS1411 aptamer for active nucleus delivery and a Förster resonance energy transfer (FRET) array for signal readout. In living cells, TP‐SA enabled monitoring of nuclear OGG1 activity, revealing distinct variations in basal enzymatic levels across various cell lines. Additionally, preliminary evaluations in bronchoalveolar lavage fluid from pneumonia patients suggested its applicability in clinical settings. Furthermore, it served as a platform for pharmacological validation, effectively assessing the effects of small‐molecule activators on OGG1. This study established a powerful framework for designing spatially specific nanoprobes, successfully enabling intranuclear imaging and in situ profiling of OGG1 activity. This advancement has transcended the boundaries of traditional biosensors, providing powerful and multi‐dimensional tools for basic biological sensing, clinical analysis in complex biological fluids, and precision medicine fields.

## Graphical abstract

The TP‐SA nanoprobe, integrating an AS1411 aptamer for nuclear delivery and a FRET array for signal amplification, enables real‐time tracking of nuclear OGG1 activity. It reveals cell‐line‐specific basal OGG1 levels and shows clinical promise using pneumonia patients’ lavage fluid. Additionally, it serves as a platform to assess small‐molecule OGG1 activators.

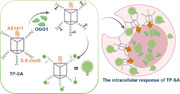

## 1 Introduction

Oxidative DNA damage, with 8‐oxoguanine (8‐oxoG) as a principal lesion, is a fundamental driver of genomic instability and disease pathogenesis [1, 2, 3, 4, 5]. As a highly mutagenic lesion that induces G:C to T:A transversions, the accurate quantification of 8‐oxoG is essential for understanding cellular stress and disease mechanisms [6, 7, 8]. However, the critical importance of monitoring 8‐oxoG dynamics in situ is contrasted by profound technical challenges. Established analytical techniques, such as DNA sequencing or chromatography‐mass spectrometry, offer high accuracy but require complex procedures involving DNA extraction, digestion, and often amplification, precluding dynamic analysis in intact cells [9, 10, 11]. Conversely, fluorescence‐based approaches for real‐time imaging are chronically hampered by a low signal recognition rate, stemming from i) spatial mismatch, as probes often fail to accumulate within the nucleus where genomic 8‐oxoG located, leading to signal dilution [12, 13]; and ii) insufficient signal modulation, characterized by high background fluorescence and a modest response upon target recognition [14]. Consequently, a technology that integrates precise subcellular targeting with a potent signal amplification strategy is urgently required to overcome these limitations and enable the rigorous, dynamic quantification of 8‐oxoG in living cells.

**SCHEME 1:**
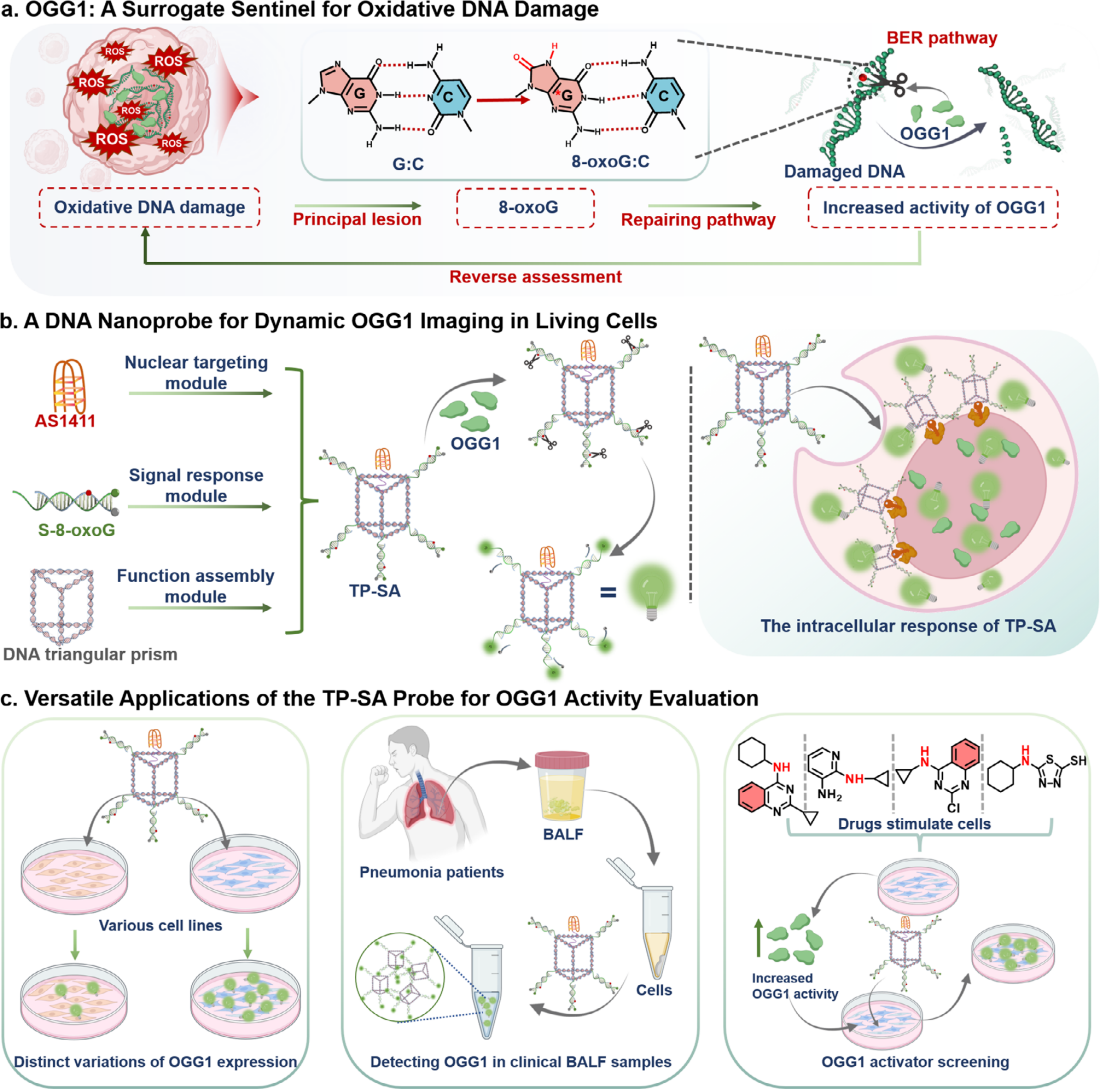
Schematic of the nuclear‐targeted OGG1 detection platform. (a) The manifestations of DNA oxidation damage and the repair process. (b) Schematic diagram of the design principle of nuclear‐targeted DNA nanoprobe. (c) The application of TP‐SA nanoprobe in investigating the distinct variations of OGG1 expression across various cell lines, providing auxiliary insights into pulmonary inflammation, and screening OGG1 activators.

To circumvent the inherent difficulties of 8‐oxoG detection, a paradigm‐shifting strategy can be proposed, based on monitoring the activity of a related repair enzyme rather than directly detecting the 8‐oxoG [15, 16]. Specifically, the activity of 8‐oxoG DNA glycosylase‐1 (OGG1) from the base excision repair (BER) pathway can be utilized [17, 18, 19]. OGG1 plays a critical role in maintaining genomic integrity by specifically recognizing and removing oxidized purines from DNA [20, 21, 22]. The catalytic action of OGG1 is correlated with the concentration of its 8‐oxoG, allowing its activity to serve as a potential surrogate for the cellular 8‐oxoG load [23, 24]. Monitoring the catalytic activity of OGG1 offers a compelling strategy to indirectly quantify 8‐oxoG, as it reframes a stoichiometric detection problem into an enzymatic assay inherently amenable to signal amplification, thereby paving the way for in situ imaging in living cells (Scheme 1a). Due to the unparalleled programmability, nanoscale spatial localization capabilities, and excellent biocompatibility, DNA nanostructures have emerged as a powerful and versatile platform in the field of subcellular bioimaging [25, 26]. However, despite these advances, achieving nuclear‐targeted imaging of DNA repair enzymes remains a formidable challenge, as most DNA nanostructures are strongly repelled by the nuclear envelope. Therefore, urgently requires a DNA nanostructure capable of simultaneously enabling active nuclear delivery and signal amplification.

Herein, a DNA‐based triangular prism nanoprobe (denoted as TP‐SA) was engineered to surmount the formidable challenges of sensitive and reliable OGG1 imaging in living cells. Two fundamental obstacles still hinder sensitive imaging of OGG1. The first one is spatial localization: accurate assessment of genomic integrity requires interrogating OGG1 activity within the nucleus, the primary reservoir of its 8‐oxoG substrate, yet conventional nanoprobes are largely thwarted by the nuclear envelope. To address this, TP‐SA was functionalized with an AS1411 aptamer, which acted as a molecular shuttle to actively traffic the nanoprobe across the nuclear membrane for targeted enrichment. The second obstacle is signal amplification: the inherently weak signal from low‐abundance enzymes is often submerged in cellular autofluorescence. To resolve this, TP‐SA was constructed on a rigid DNA triangular prism scaffold precisely patterned with a synergistic array of FRET units, an architecture that converted a single enzymatic cleavage into an amplified fluorescence response (Scheme 1b). This integrated design, which simultaneously solved the delivery and amplification problems, enabled the ultrasensitive monitoring of nuclear OGG1 dynamics under chemical stress and served as a high‐throughput platform for screening OGG1‐activating drugs (Scheme 1c).

## 2 Results and Discussion

### 2.1 Design and Characterization of the TP‐SA Nanoprobe

**FIGURE 1:**
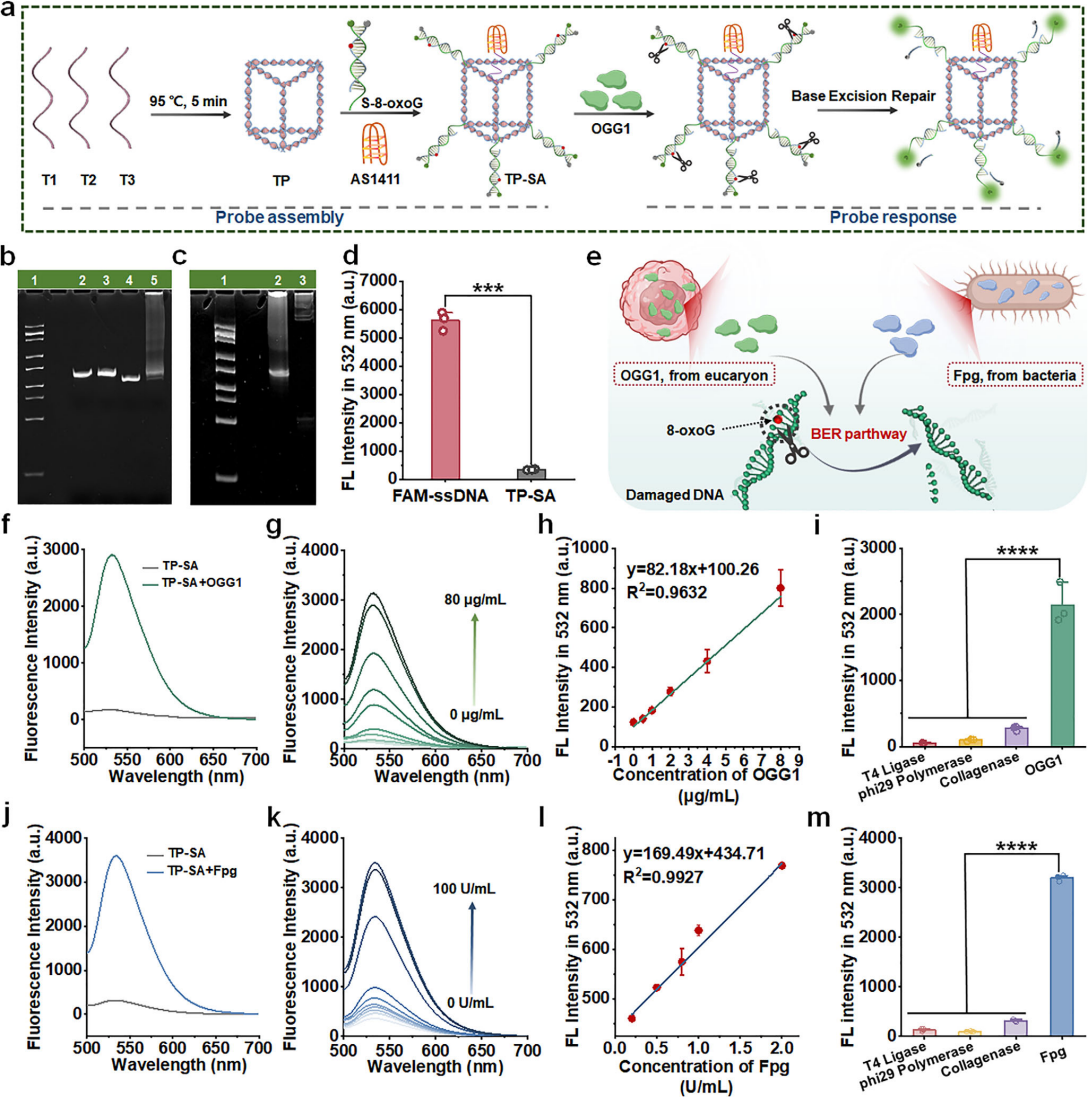
(a) Schematic illustration of the assembly and OGG1‐responsive mechanism of the TP‐SA. (b) Representative 12% PAGE analysis of the TP scaffold assembly: lane 1, 25–500 bp DNA marker; lanes 2–4, individual strands T1, T2, and T3; lane 5, assembled TP scaffold. (c) Representative 12% PAGE analysis of TP‐SA nanoprobe: lane 1, DNA marker; lane 2, TP scaffold; lane 3, assembled TP‐SA. (d) Fluorescence quantification of FAM‐ssDNA and TP‐SA assembly. (e) Schematic illustration of the DNA repair mechanisms by OGG1 and Fpg. (f) Fluorescence emission spectra of TP‐SA in response to OGG1. (g) Fluorescence response spectra of TP‐SA to different concentrations of OGG1. (h) Standard curve for OGG1 activity detection derived from the data in panel (g) (*n* = 3). (i) Specificity investigation of TP‐SA for OGG1 (*n* = 3). (j) Fluorescence emission spectra of TP‐SA in response to Fpg. (k) Fluorescence response spectra of TP‐SA to different concentrations of Fpg. (l) Standard curve for Fpg activity detection derived from the data in panel (k) (*n* = 3). (m) Specificity investigation of TP‐SA for Fpg (*n* = 3).

A DNA triangular prism nanoprobe, designated TP‐SA, was engineered to enable the sensitive detection of OGG1 activity by integrating a nuclear‐targeting module with a signal amplification system (Figure 1a, sequences in Table S1). The signal amplification core was constructed by anchoring five copies of a FRET‐based substrate duplex (S‐8‐oxoG) onto the vertices of a rigid DNA triangular prism (TP) scaffold. Each S‐8‐oxoG duplex comprised a FAM‐labeled reporter strand and a quencher strand modified with BHQ1 and containing the OGG1‐specific 8‐oxoG lesion. To facilitate spatial enrichment within the nucleus, the nucleolin‐binding aptamer AS1411 was conjugated to the remaining vertex of the TP scaffold.

The stepwise assembly of the nanoprobe was validated by 12% polyacrylamide gel electrophoresis (PAGE). The successful formation of the TP scaffold from its three constituent single strands (T1, T2, T3) was evidenced by the appearance of a single, slower‐migrating band compared to the individual strands (Figure 1b, lane 5). Subsequent incubation with the S‐8‐oxoG duplexes and the AS1411 aptamer resulted in a final TP‐SA construct with a significantly higher molecular weight, causing its retention within the loading well (Figure 1c, lane 3). This pronounced shift in electrophoretic mobility confirmed the successful of all components into the final integrated nanostructure. Furthermore, a characterization of the morphology of TP‐SA was conducted using atomic force microscopy (AFM), and the scale size confirmed its monodispersity (Figure S1).

The nanoprobe's capacity for high‐contrast imaging relies on a low‐background “off” state, which was first confirmed by fluorescence spectroscopy. The fluorescence of the fully assembled TP‐SA nanoprobe was suppressed by more than 90% relative to the unquenched S‐8‐oxoG substrate, demonstrating efficient FRET‐based quenching within the assembled nanostructure (Figure 1d and Figure S2). Two DNA glycosylases with similar functions but different sources, OGG1 from eukaryotes and formamidopyrimidine DNA glycosylase (Fpg) from bacteria, both could mediate the recognition and excise of 8‐oxoG oxidative damage, were chosen for the in vitro response of TP‐SA nanoprobe (Figure 1e) [27]. Incubation with OGG1 (Figure 1f and Figure S3) or Fpg (Figure 1j and Figure S4) resulted in the enzymatic excision of the 8‐oxoG lesion and subsequent release of the quencher‐bearing strand, leading to a robust restoration of FAM fluorescence. The fluorescence intensity exhibited a concentration‐dependent increase in the presence of escalating concentrations of OGG1 (Figure 1g and Figure S5) or Fpg (Figure 1k and Figure S6). The fluorescence response was linear over an OGG1 concentration range from 0 to 8 µg/mL (R^2^ = 0.9632), with a calculated limit of detection (LOD) of 0.16 µg/mL (Figure 1h). While for Fpg, linear results ranged from 0.2 to 2.0 U/mL (R^2^ = 0.9927), with a LOD of 0.07 U/mL (Figure 1l). At the same time, a comparison was made between TP‐SA and the existing representative methods (Table S2). Furthermore, the nanoprobe's response was highly specific, as negligible fluorescence change was observed in the presence of other non‐target nucleases, including T4 ligase, phi29 polymerase, and collegenase, underscored its excellent enzymatic selectivity in OGG1 (Figure 1i and Figure S7) or Fpg (Figure 1m and Figure S8). To further verify that the fluorescence response of TP‐SA nanoprobe was indeed attributable to OGG1, a nanoprobe without 8‐oxoG (denoted as TP‐NSA) was constructed as a control. The results were shown in Figure S9, in the absence of 8‐oxoG, TP‐NSA did not exhibit a noticeable fluorescent response. While the fluorescent signal of TP‐SA nanoprobe was significantly restored. This indicated that TP‐SA reflected the OGG1‐specific repair process rather than degradation caused by non‐specific nucleases.

Collectively, these characterizations provided compelling evidence for the successful engineering of the TP‐SA nanoprobe. The data confirmed the assembly of the integrated architecture and validated its core signaling mechanism, which featured a low‐background “off” state and a specific, high‐gain “on” state. This established the nanoprobe's foundational capability to achieve a signal amplification required for sensitive intracellular analysis.

### 2.2 Quantitative Interrogation of Intracellular OGG1 Activity With TP‐SA Nanoprobe

**FIGURE 2:**
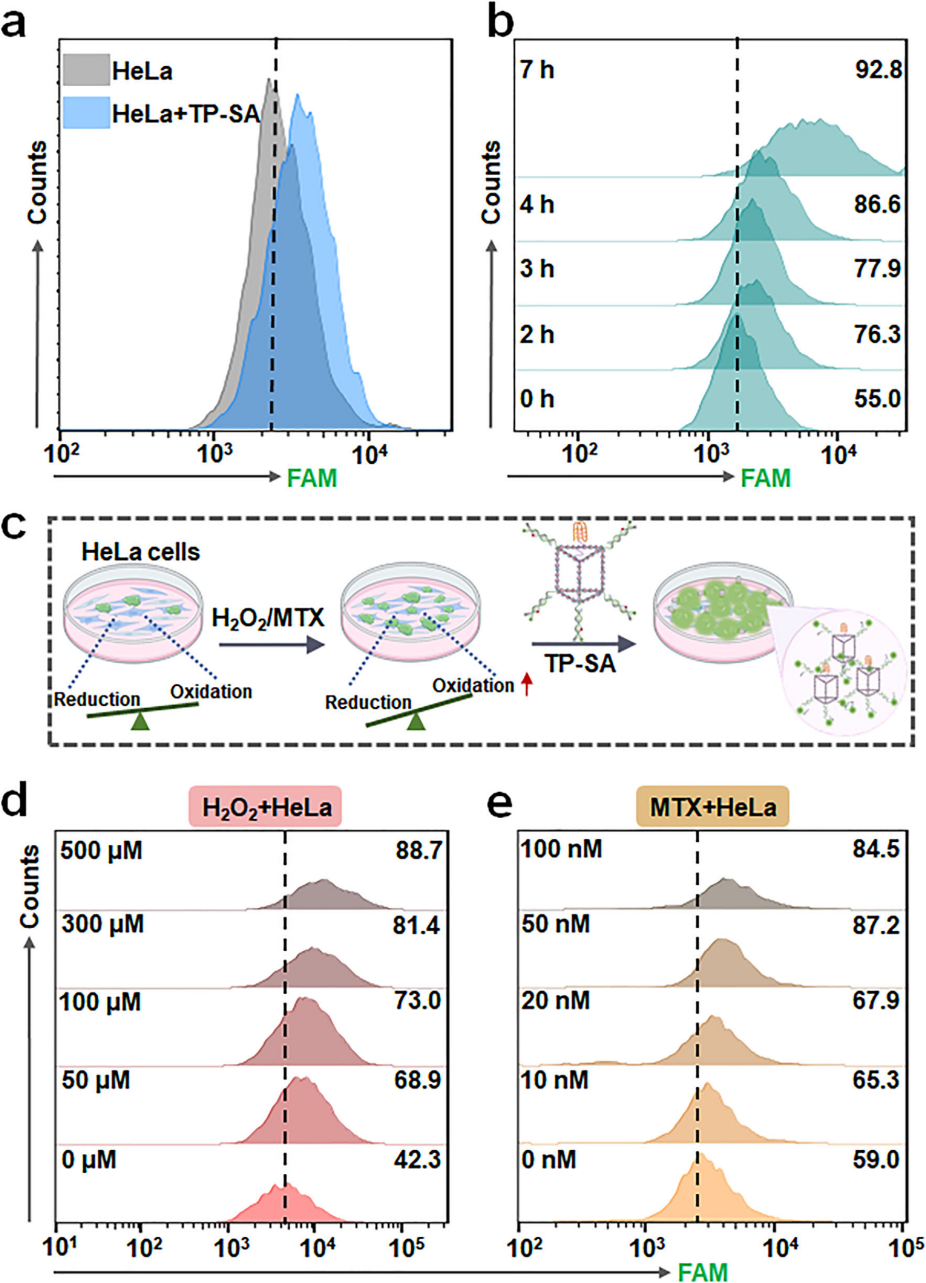
(a) Representative flow cytometry histograms of OGG1 activity in HeLa cells treated without or with TP‐SA. (b) Flow cytometry analysis of TP‐SA nanoprobe response in HeLa cells at various incubation times. (c) Diagram illustrating the process of induced oxidative stress. (d) Dose‐dependent OGG1 activity in H_2_O_2_‐stimulated HeLa cells and (e) in MTX‐treated cells, as measured by flow cytometry.

For oxidative DNA damage in eukaryotic cells, OGG1 was selected as the target for subsequent research. Here, the capacity of TP‐SA for the quantitative interrogation of OGG1 activity within the intracellular environment was systematically characterized. Initial validation was conducted in HeLa cells, one of the most extensively characterized human cell lines for studying OGG1‐initiated BER [28, 29]. The toxicity of the TP‐SA nanoprobe was first evaluated on HeLa cells. The results showed that with TP‐SA concentration up to 500 nm, the probes exhibited virtually no toxicity (Figure S10). Incubation of Hela cells with TP‐SA resulted in a marked elevation in the FAM‐positive population, from a baseline of 59.7% in untreated controls to 81.9%, as quantified by flow cytometry (Figure 2a). This finding substantiated the nanoprobe's efficacy in penetrating the cell and reporting basal enzymatic activity. To establish an optimized assay protocol, the kinetics of the nanoprobe's response were investigated. A time‐course analysis demonstrated that the percentage of FAM‐positive cells increased progressively, reaching a plateau at approximately 86.6% after 4 h of incubation (Figure 2b).

To rigorously validate the specificity of TP‐SA toward endogenous OGG1, we performed siRNA‐mediated knockdown experiments. The successful depletion of OGG1 was first confirmed via western blotting, and semi‐quantitative analysis revealed that its protein expression was reduced by approximately 80% (Figure S11). The TP‐SA nanoprobe was then incubated with OGG1‐knockdown HeLa cells (HeLa OGG1 KD). As shown in Figure S12a, the FAM‐positive cell population was significantly reduced in the OGG1 KD group compared to the control group. Consistently, confocal microscopy revealed a marked attenuation of intracellular green fluorescence upon OGG1 depletion (Figure S12b). These results confirmed that the TP‐SA signal in living cells was primarily driven by the catalytic activity of OGG1 and was not confounded by other BER glycosylases.

To evaluate whether TP‐SA could faithfully report on modulations of intracellular OGG1 activity under oxidative stress, we challenged HeLa cells with two distinct oxidative stimuli (Figure 2c). First, cells were treated with exogenous H_2_O_2_, a widely employed inducer of intracellular oxidative stress [30]. Flow cytometry revealed a dose‐dependent enhancement of FAM fluorescence intensity with increasing H_2_O_2_ concentrations (0–500 µm), indicative of progressively elevated OGG1 enzymatic activity within the cells (Figure 2d). Next, to assess the nanoprobe's capacity to detect OGG1 activity changes elicited by pharmacological agents, cells were treated with methotrexate (MTX), a folate antagonist widely used in chemotherapy and immunosuppression, has been reported to induce intracellular ROS accumulation and oxidative stress, thereby triggering DNA repair responses [31]. Consistent with the H_2_O_2_ results, MTX treatment produced a dose‐dependent increase in FAM fluorescence across the tested concentration range (0–100 nm) (Figure 2e), confirming the nanoprobe's ability to detect OGG1 activity changes induced by pharmacologically mediated oxidative stress.

Collectively, these cellular assays demonstrated that TP‐SA functions as a high‐fidelity molecular nanoprobe, capable of not only quantifying basal OGG1 activity but also dynamically tracking its upregulation in response to both direct oxidative insults and indirect pharmacological triggers. These results underscored the nanoprobe's significant potential as a precise tool for elucidating the roles of OGG1 in complex biological processes.

### 2.3 AS1411‐Mediated Nuclear Targeting Maximized Signal From the Primary OGG1 Compartment

**FIGURE 3:**
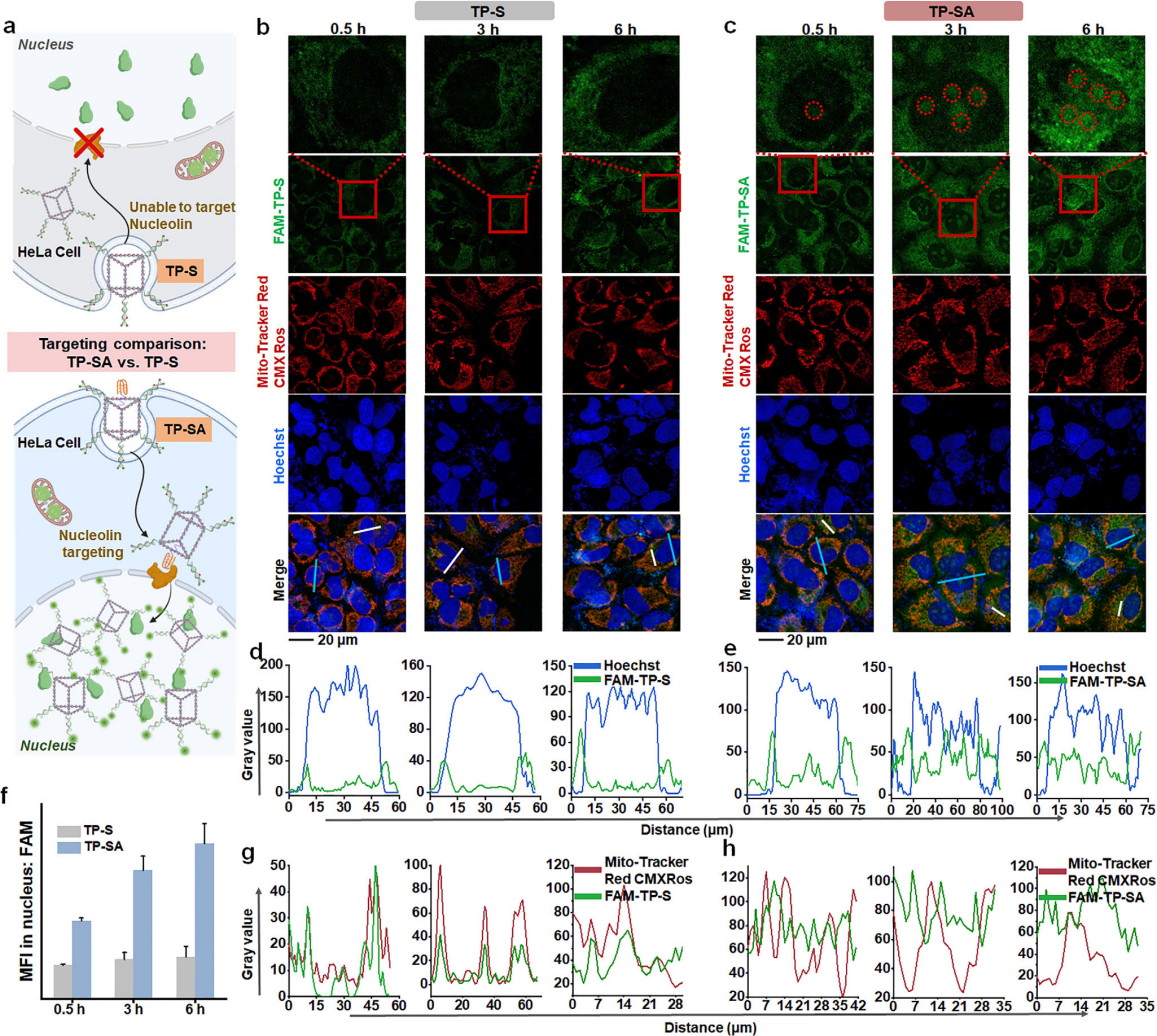
(a) Schematic illustration of nuclear‐targeting OGG1 detection via TP‐SA. Confocal microscopy images of HeLa cells treated with TP‐S (b) and TP‐SA (c) at various time points. Blue line: The co‐localization of FAM‐TP‐S or FAM‐TP‐SA with the nucleus; white line: the co‐localization of FAM‐TP‐S or FAM‐TP‐SA with mitochondria. Co‐localization analysis between green fluorescence and nuclear staining in cells treated with TP‐S (d) and TP‐SA (e). (f) Quantification of the green fluorescence intensity derived from (b,c) in the HeLa cell nucleus. Co‐localization of green fluorescence with mitochondrial staining (MitoTracker Red) in TP‐S (g) and TP‐SA (h) treated groups.

Having established the nanoprobe's capacity to report on OGG1 activity dynamics, we next sought to validate the core design principle underpinning its precision: active targeting to the nucleus, the principal subcellular compartment for OGG1‐mediated DNA repair. The nuclear targeting of TP‐SA relied on the AS1411 aptamer, a G4‐forming oligonucleotide that binds nucleolin (NCL) with high affinity. NCL was fundamentally a nuclear and nucleolar protein. In many cancer cell types, however, NCL was aberrantly overexpressed on the cell surface and undergoes continuous shuttling between the plasma membrane, cytoplasm, and nucleus. It should be noted that the efficiency of this nuclear delivery route might varied across cell types depending on nucleolin expression levels and intracellular trafficking dynamics [32]. Upon binding surface NCL, the AS1411–NCL complex is internalized via macropinocytosis and subsequently trafficked to the nucleus through NCL's intrinsic NLS‐mediated transport pathway [33, 34, 35, 36]. To rigorously assess the impact of this strategy, a comparison was performed in HeLa cells, nucleus‐targeting TP‐SA nanoprobe was benchmarked against its non‐targeted counterpart, TP‐S, which lacked the AS1411 aptamer (Figure 3a).

Confocal images revealed a stark divergence in the subcellular fate and signaling behavior of the two nanoprobes. While both nanoprobes exhibited a time‐dependent increase in fluorescence, indicative of successful enzymatic activation, their spatial distributions were fundamentally disparate (Figure 3b,c). The green fluorescence signal from TP‐SA exhibited pronounced co‐localization with the Hoechst‐stained nucleus, verifying its precise delivery to this target organelle (Figure 3e). Conversely, the TP‐S signal showed no significant nuclear overlap (Figure 3d). The signal from the non‐targeted TP‐S nanoprobe remained diffusely distributed throughout the cytoplasm and was largely excluded from the nucleus. By contrast, the TP‐SA nanoprobe underwent progressive and specific accumulation within the nucleus, forming intense fluorescent foci. To confirm this, fluorescence analysis revealed that after 6 h of incubation, the nuclear fluorescence intensity in TP‐SA‐treated cells was approximately 3.7 times higher than that in TP‐S‐treated cells (Figure 3f). This result directly demonstrated that active targeting could dramatically enhance the signal within the primary organelle of interest.

Co‐localization studies provided unequivocal confirmation of the nanoprobe's targeting fidelity. Further investigation using a mitochondrial marker (MitoTracker Red) elucidated the fate of the non‐targeted nanoprobe. TP‐S was found to be predominantly sequestered within mitochondria, a known secondary location for OGG1 activity [37, 38], which accounts for its diffuse cytoplasmic staining pattern (Figure 3g). Intriguingly, the TP‐SA nanoprobe also displayed partial co‐localization with mitochondria, indicating its capacity to report on the enzyme in this compartment as well (Figure 3h). This sophisticated dual‐localization behavior, characterized by preferential nuclear accumulation, highlighted the nanoprobe's advanced design, enabling enhanced detection in the primary OGG1 compartment while retaining sensitivity to enzymatic activity in secondary locales.

### 2.4 The Revelation of the Intrinsic OGG1 Activity through TP‐SA

**FIGURE 4:**
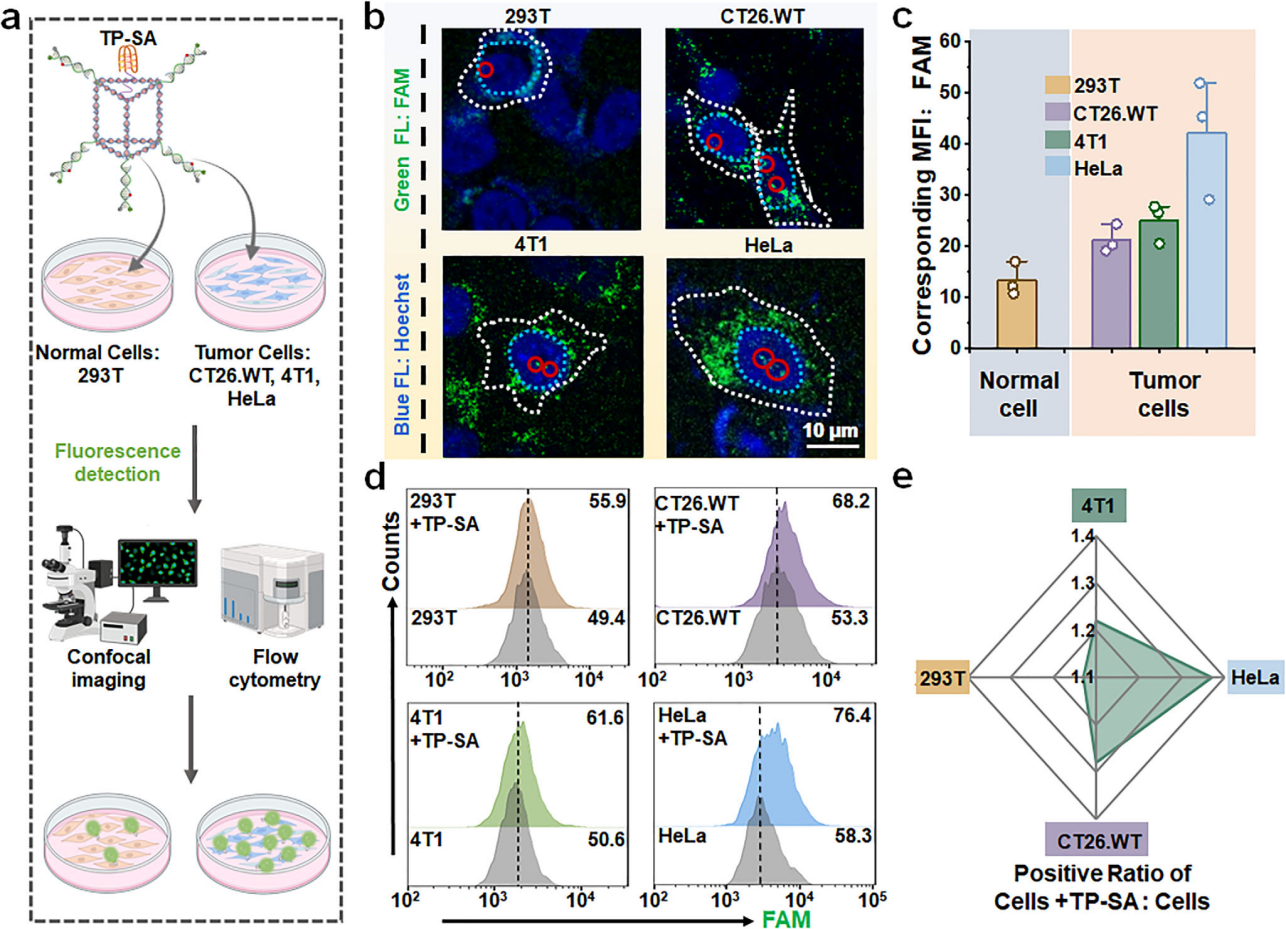
(a) Schematic of the procedure for detecting OGG1 expression in different cell lines using the TP‐SA nanoprobe. (b) Representative confocal microscopy images of various cell lines (293T, CT26.WT, 4T1, HeLa) after 4 h incubation with the TP‐SA nanoprobe (green: FAM; blue: Hoechst). (c) Quantification of mean nuclear green fluorescence intensity derived from the images in (a). (d) Flow cytometry histograms (FAM channel) showing the response of different cell lines to TP‐SA treatment for 4 h (*n* = 3). (e) Radar chart illustrating the signal‐to‐background ratio (SBR) values calculated from the flow cytometry data presented in (d).

Having validated the superior targeting and signaling fidelity of TP‐SA, we deployed it to the variations of DNA repair capacity across various cell lines. The OGG1 activity was first imaged in panels of four distinct cell lines, human embryonic kidney cells (293T) and three cancer lines of mouse colon carcinoma (CT26.WT), mouse breast cancer (4T1), and HeLa (Figure 4a). Following 4 h incubation with TP‐SA, confocal images unveiled a spectrum of OGG1 activity (Figure 4b). HeLa cells exhibited the most intense fluorescence, indicative of the highest basal OGG1 levels, followed by CT26.WT and 4T1 cells. In stark contrast, normal cell 293T displayed the lowest signal (Figure 4c). We further corroborated these observations by flow cytometric analysis. In Figure 4d, the signal‐to‐background ratio (SBR) in the FAM channel was determined by using the autofluorescence peak of untreated cells from each line as a reference. As summarized in the radar chart (Figure 4e), the SBR value for HeLa cells (1.37) was significantly higher than those of other cell lines (293T: 1.13; 4T1: 1.22; CT26.WT: 1.28). The differences in the expression of OGG1 between normal cells and tumor cells, besides the fact that tumor cells generally exhibited a more pronounced oxidative stress state, it might also be due to the fact that tumor cells express more NCL [39, 40]. In addition, it should be noted that the specific ranking of OGG1 activity among these cell lines was not absolute, as basal OGG1 activity in various cell lines could be influenced by a constellation of variables, including cell passage number, culture conditions, serum batch, and the inherent genetic drift accumulated during long‐term propagation of established cell lines [41, 42].

### 2.5 Translational Validation of TP‐SA in Clinical Samples From Pneumonia Patients

**FIGURE 5:**
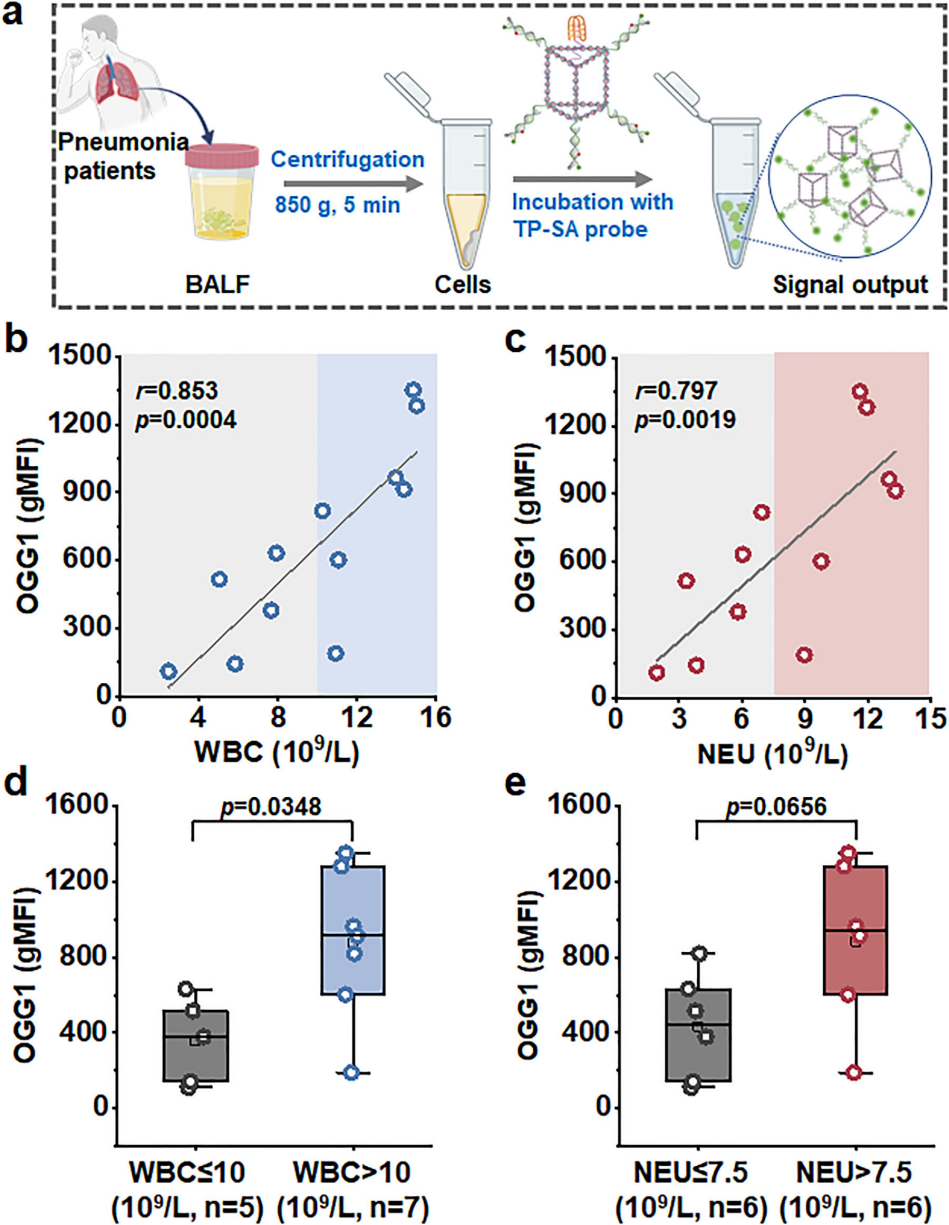
(a) Workflow for detecting OGG1 activity in BALF‐derived cells using the TP‐SA nanoprobe. Linear regression analysis evaluating the relationship between BALF OGG1 gMFI and the clinical data of WBC (b) and NEU (c). Quantitative comparison of OGG1 levels in patient subgroups stratified by the clinical thresholds for WBC (d) and NEU (e).

Beyond validating probe performance, we conducted an exploratory analysis to assess whether OGG1 activity in bronchoalveolar lavage fluid (BALF) ‐derived cells might serve as a potential auxiliary clinical indicator. It has been reported that mice lacking the gene encoding OGG1 exhibit resistance to inflammation [43]. Moreover, OGG1 has been implicated as playing a potentially critical role in pulmonary infection and other inflammatory lung diseases [44, 45]. Small‐molecule‐mediated OGG1 inhibition has been shown to attenuate pulmonary inflammation and lung fibrosis in a murine lung fibrosis model [46], and the selective OGG1 inhibitor TH5487 markedly reduced neutrophil infiltration in a pulmonary inflammation model using C57BL/6J mice [47]. Collectively, these studies suggested that OGG1 activity might, at least in part, reflect the degree of local oxidative and inflammatory burden, and that its potential value as an inflammatory biomarker in pneumonia patients warranted investigation. To this end, BALF from a cohort of 12 pneumonia patients was selected as the testing substrate. Pneumonia‐associated airways were characterized by intense local inflammation and elevated oxidative stress, producing BALF samples that contained a diverse array of potential interferents, including mucoproteins, proteases, and cell debris. Cells were isolated from BALF via centrifugation, and intracellular OGG1 activity was quantified by flow cytometry using the TP‐SA nanoprobe (Figure 5a and Figure S13).

To rigorously evaluate the clinical consistency between BALF OGG1 activity and systemic White Blood Cell (WBC) and Neutrophil (NEU) data (Table S3), statistical analyses were performed. Spearman rank correlation analysis revealed a positive correlation between the BALF OGG1 geometry mean fluorescence intensity (gMFI) and WBC counts (Spearman's *r* = 0.853, *p* = 0.0004) (Figure 5b). A similar correlation was established with NEU counts (Spearman's *r* = 0.797, *p* = 0.0019) (Figure 5c). Furthermore, to assess the feasibility of utilizing BALF OGG1 as a companion diagnostic biomarker, the clinical samples were stratified based on standard diagnostic thresholds for inflammation (WBC > 10 × 10^9^/L and NEU > 7.5 × 10^9^/L). Mann‐Whitney *U* tests were subsequently applied to evaluate the discriminatory capacity of the nanoprobe. As illustrated in Figure 5d, the OGG1 gMFI was significantly elevated in the high‐WBC group compared to the normal‐WBC group (*p* = 0.0348), demonstrating potential clinical stratification. For the NEU‐based stratification (Figure 5e), an upward trend in OGG1 activity was observed in the high‐NEU cohort. Although this divergence exhibited marginal statistical significance (*p* = 0.0656), likely constrained by the limited sample size following rigorous quality control. It was important to emphasize that the results presented in this section were intended primarily to demonstrate the analytical capability and robustness of the TP‐SA nanoprobe, rather than to establish definitive clinical diagnostic conclusions. Therefore, the clinical significance of OGG1 as a standalone or auxiliary biomarker remains to be established through large‐scale, multi‐center clinical investigations with rigorous stratification.

### 2.6 Screening of OGG1 Activators Using the TP‐SA Nanoprobe

**FIGURE 6:**
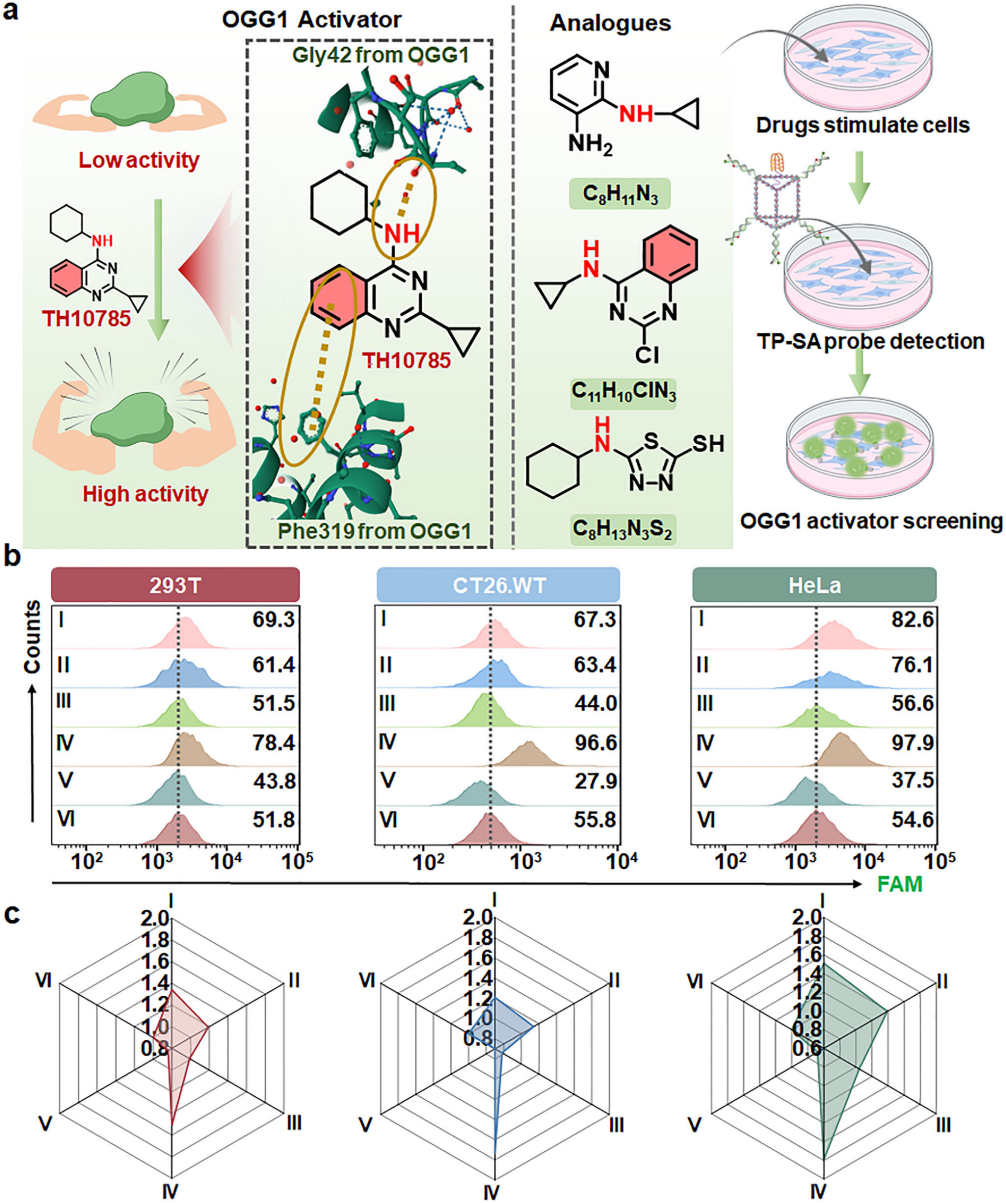
(a) Schematic representation of the interaction between OGG1 activators and the enzyme's active site. The protein structure of OGG1 was derived from the RCSB database. (b) Flow cytometry histograms (FAM channel) depicting the effects of various drug treatments on OGG1 activity across three cell lines (293T, CT26.WT, HeLa). A unified threshold, set at the autofluorescence peak of untreated cells, was used to quantify the percentage of FAM^+^ cells. (c) Radar plot illustrating drug response efficacy derived from the data in (b) (I: C_8_H_11_N_3_; II: C_11_H_10_ClN_3_; III: C_8_H_13_N_3_S_2_; IV: TH10785; V: TH5487; VI: untreated cells+TP‐SA). The metric represents the ratio of the percentage of FAM^+^ cells in drug‐treated groups to that in blank controls.

Another critical application of a bio‐molecular nanoprobe lied in its utility for pharmacological screening. To validate TP‐SA's utility as an evaluation platform, we employed the OGG1 activator TH10785 as a positive control and the inhibitor TH5487 as a negative control (Figure 6a). To evaluate the reliability of the TP‐SA nanoprobe in detecting changes in OGG1 activity under pharmacological intervention, we conducted dose‐response experiments in HeLa cells. Cells were treated with five different concentrations of either TH10785 or TH5487. Subsequently, OGG1 activity was measured using TP‐SA nanoprobe in combination with flow cytometry. As shown in Figure S14a, TH10785 induced a dose‐dependent increase in OGG1 activity; conversely, TH5487 dose‐dependently inhibited OGG1 activity (Figure S14b). These dose‐response curves collectively confirmed that the TP‐SA nanoprobe could stably detect the activation and inhibition states of OGG1 over a wide concentration range.

Guided by the established binding mode of TH10785, we verified the applicability of TP‐SA as a drug screening platform. The interaction of TH10785 with OGG1 was stabilized by key structural motifs, including *π–π* stacking between its quinazoline core and phenylalanine‐319 (Phe‐319), and a hydrogen bond interaction involving a nitrogen linker atom and glycine‐42 (Gly‐42) [48]. Based on these critical features, the aromatic ring system and the nitrogen‐containing heterocycle, three structural analogs were selected for potential activator screening: N_2_‐Cyclopropylpyridine‐2,3‐diamine (C_8_H_11_N_3_), 2‐Chloro‐N‐cyclopropylquinazolin‐4‐amine (C_11_H_10_ClN_3_), and 5‐(Cyclohexylamino)‐3H‐1,3,4‐thiadiazole‐2‐thione (C_8_H_13_N_3_S_2_) (Figure 6a).

Using the flow cytometry‐based TP‐SA assay, the effects of these compounds on OGG1 activity was systematically evaluated in a panel of normal (293T) and cancer (CT26.WT, HeLa) cell lines (Figure 6b). CCK‐8 assays confirmed that, under experimental conditions, cell viability in all treatment groups was >70% (Figure S15), none of the compounds significantly reduced cell viability, thereby ruling out indirect effects caused by cytotoxicity. Among these structural analogs, C_8_H_11_N_3_ induced an increase in OGG1 activity, although its effect was less potent than that of the positive control, TH10785. In contrast, C_8_H_13_N_3_S_2_ elicited only a minimal change. This differential activity likely reflected the higher degree of structural conservation between C_8_H_11_N_3_ and activator TH10785. Quantitative analysis of the FAM^+^ cell population further substantiated these findings (Figure 6c). For instance, stimulation with TH10785 nearly doubled OGG1 activity in CT26.WT cells, while the newly identified C_8_H_11_N_3_, induced a significant 1.51‐fold increase in activity in HeLa cells.

In short, these results provided definitive pharmacological validation for the application of the TP‐SA nanoprobe in living cells and establish this flow cytometry‐based method as a powerful, scalable, and high‐throughput platform for the discovery and characterization of novel OGG1 activators.

## 3 Conclusion

In summary, we have rationally designed TP‐SA, a nuclear‐targeted DNA triangular prism nanoprobe engineered for the sensitive detection of OGG1 activity. This nanoprobe integrated an AS1411 aptamer for active spatial enrichment within the nucleus with a FRET array for signal amplification. Such a bifunctional design effectively overcame the persistent challenges of nanoprobe‐target spatial discordance and insufficient signal gain inherent to subcellular analysis. The versatility and robustness of the TP‐SA platform were demonstrated by its capacity to dynamically monitor OGG1 activity in response to oxidative and pharmacological stimuli in living cells. Furthermore, it enabled the delineation of enzymatic variations across distinct cell lines. Critically, the detection potential of TP‐SA was confirmed using BALF from pneumonia patients. Collectively, this work established a generalizable design principle and framework for engineering DNA nanoprobes capable of probing subcellular enzymatic activity.

## 4 Experimental Section

### 4.1 Materials

All oligonucleotides utilized in this study (Table S1) were chemically synthesized and purified through high‐performance liquid chromatography (HPLC) by Sangon Biotech (Shanghai) Co., Ltd. DNA marker (25–500 bp) was purchased from the same supplier. OGG1 was provided by MedChemExpress LLC. Fpg was provided by Beijing New England Biotechnology Laboratory Company. Hoechst 33258, Lyso‐Tracker Red, and C_8_H_11_N_3_ were purchased from Beyotime Biotechnology Co., Ltd. (Shanghai, China). TH10785 was procured from Boka Co., Ltd. (Shanghai, China). TH5487 and C_11_H_10_ClN_3_ were provided by Shanghai Macklin Biochemical Co., Ltd. C_8_H_13_N_3_S_2_ was provided by Bide Pharmatech Co., Ltd. Dulbecco's phosphate‐buffered saline (DPBS), RPMI‐1640 medium, and Dulbecco's modified Eagle medium (DMEM) were obtained from Jingrun Biotechnology Co., Ltd. (Hangzhou, China). Fetal bovine serum (FBS) was procured from Sorfa Life Science Research Co., Ltd. (Deqing County, China). Deionized water (18.2 MΩ·cm) prepared using a water purification system was used for all experiments.

### 4.2 Apparatus

Fluorescence spectra were acquired on an F‐7000 fluorescence spectrophotometer (Hitachi, Japan). SDS‐PAGE was visualized and analyzed with a ChemiDoc XRS^+^ imaging system (Bio‐Rad, USA). Flow cytometric analysis was performed on an Attune NxT Flow Cytometer (Thermo Fisher Scientific, USA) equipped with dual laser systems (488 nm). Cellular fluorescence imaging was acquired through a scanning confocal microscope (Carl Zeiss AG, Germany) under standardized parameters: 40× water‐immersion objective. The characterization of the TP‐SA structure was obtained using Cypher VRS atomic force microscope (Asylum Research, USA).

### 4.3 Preparation of the FRET‐Based Substrate Duplex (S‐8‐oxoG)

An equimolar mixture of a FAM‐labeled single‐stranded DNA (S1‐A‐E) and a BHQ1‐labeled ssDNA (S2) containing the 8‐oxoG lesion was prepared in 1× Tris‐Mg^2+^ buffer. The mixture was heated to 95°C for 5 min and then gradually cooled to room temperature over 60 min to facilitate annealing, yielding the S‐8‐oxoG duplex.

### 4.4 Assembly of the Nanoprobes

The TP scaffold was synthesized by annealing an equimolar mixture of its three constituent ssDNAs (T1, T2, and T3) using the identical thermal annealing protocol described above. To construct the TP‐SA, the pre‐formed TP scaffold, the S‐8‐oxoG duplex, and the AS1411 aptamer were incubated at a molar ratio of 1:5:1 at 37°C for 1 h. A non‐targeted control nanoprobe (TP‐S) was synthesized following an identical procedure, with the omission of the AS1411 aptamer. The synthesis process for TP‐NSA nanoprobe was the same as described above, with the exception that the chain lacked 8‐oxoG.

### 4.5 PAGE Assay

The stepwise assembly of the TP scaffold and the final TP‐SA nanoprobe was verified by 12% native PAGE. Aliquots of the individual DNA strands (T1, T2, T3), the assembled TP scaffold, and the final TP‐SA nanoprobe were loaded onto the gel. Electrophoresis was conducted in 1×TBE buffer at a constant 120 V for 90 min. Following electrophoresis, the gel was stained for 30 min with GelRed nucleic acid dye and visualized using a ChemiDoc XRS^+^ imaging system.

### 4.6 AFM Imaging

The mica surface was first treated with a 0.1% APTES aqueous solution for 10 min, then thoroughly rinsed and dried with nitrogen gas. Subsequently, a 1 nm sample solution was incubated on the mica slide for 5 min to allow for fixation. Next, the sample was scanned using a Cypher VRS atomic force microscope in “AC Water Morphology” mode with a BL‐AC40TS probe.

### 4.7 Spectroscopic and Enzymatic Assays

The successful assembly and fluorescence quenching of the TP‐SA nanoprobe were confirmed by fluorescence spectroscopy. The emission spectrum of a 200 nm TP‐SA solution was compared to that of an equimolar concentration of the unquenched FAM‐labeled ssDNA. To characterize the enzymatic responsiveness, OGG1 (60 µg/mL) was added to a 200 nm solution of TP‐SA and incubated at 37°C for 3 h. The recovery of fluorescence was monitored by recording the emission spectrum from 500 to 700 nm, with an excitation wavelength of 488 nm. The response validation experiment for the Fpg enzyme (100 U/mL) followed the same procedure as described above.

### 4.8 Cell Culture and Maintenance

All cell lines were maintained at 37°C in a 5% CO_2_ incubator. CT26.WT cells were cultured in RPMI‐1640 medium. 4T1, HeLa, and 293T cells were cultured in DMEM. All media were supplemented with 10% (v/v) FBS and 1% (v/v) penicillin‐streptomycin.

### 4.9 Cytotoxicity Assay

To assess cytotoxicity, 293T, CT26.WT, and HeLa cells were seeded into a 96‐well plate at a density of 1 × 10^4^ cells/well. After 24 h of incubation at 37°C, the culture medium was replaced with medium containing different concentrations of small‐molecule drugs (0–60 µm) or TP‐SA nanoprobes (0–500 nm). Cytotoxicity was determined using the standard CCK‐8 assay. Each experiment was repeated 3 times to ensure reproducibility of the results.

### 4.10 SiRNA‐Mediated Silencing of the OGG1 Gene

siRNA was used to downregulate OGG1 gene expression; the sequence information is shown in Table S1. In this experiment, a 100 nm siRNA concentration was selected for transfection to mediate OGG1 gene silencing in cells. First, the double‐stranded siRNA (siOGG1) for the experimental group and the double‐stranded siRNA (siControl) for the control group were centrifuged at low speed to remove excess powder. Each was then diluted with a specified volume of sterile, nuclease‐free water to an initial concentration of 25 µm and aliquoted for use. Cells in good growth condition were selected and seeded into 6‐well cell culture plates to adhere overnight. When cell density reached 40%–50%, transfection was performed according to the manufacturer's instructions for Lipofectamine 2000.48 h after transfection, cells from each well were collected into new centrifuge tubes, labeled accordingly, and stored at −80°C.

### 4.11 Western Blot Assay

The cells were lysed using RIPA buffer supplemented with a complete protease inhibitor cocktail. The resulting lysate was centrifuged at 12,000× g for 10 min to collect the proteins, and the protein concentration was determined using a bicinchoninic acid (BCA) protein assay kit. Subsequently, the protein extracts were separated on a 12% SDS‐PAGE and transferred onto a polyvinylidene difluoride (PVDF) membrane by electrotransfer. After incubation with primary antibodies overnight at 4°C, followed by incubation with appropriate horseradish peroxidase (HRP)‐conjugated secondary antibodies for 1 h at 4°C, protein detection was performed and visualized using a ChemiDoc XRS^+^ imaging system.

### 4.12 Flow Cytometry Analysis of Intracellular OGG1 Activity

Cells were seeded into 12‐well plates at a density of 5 × 10^4^ cells/well and cultured until adherent. The culture medium was then replaced with fresh medium containing 200 nm TP‐SA, and incubated for another 2, 3, 4, and 7 h. Following incubation, cells were washed with DPBS, harvested by trypsinization, and collected by centrifugation at 300× g for 5 min at 4°C. The cell pellet was washed twice with pre‐chilled DPBS and resuspended in 500 µL of DPBS for analysis. Data were acquired on an Attune NxT Flow Cytometer using the BL1 channel (Ex: 488 nm) to detect FAM fluorescence. Data analysis was performed using FlowJo software (v10.8). Untreated, probe‐free cells served as a negative control to establish a fluorescence gate for identifying the FAM‐positive population.

### 4.13 Confocal Laser Scanning Microscopy (CLSM) and Image Analysis

HeLa cells were seeded in 6‐well plates containing glass coverslips at a density of 1 × 10^5^ cells/well and cultured for 24 h. The cells were then incubated with 200 nm TP‐SA for 0.5, 3, and 6 h at 37°C. For nuclear visualization, cells were subsequently stained with 5 µg/mL Hoechst 33258 for 30 min in the dark. After each incubation step, cells were washed twice with DPBS. Imaging was performed on a Carl Zeiss AG CLSM equipped with a 40× water‐immersion objective. Fluorescence signals were acquired using the following settings: the green channel (for TP‐SA) was excited at 488 nm and emission was collected between 500 and 550 nm; the blue channel (for Hoechst 33258) was excited at 405 nm and emission was collected between 430 and 600 nm; the red channel (for Lyso‐Tracker Red) was excited at 594 nm and emission was collected between 620 and 800 nm.

Quantitative image analysis was conducted using ImageJ software. Nuclear regions of interest (ROIs) were defined based on the Hoechst signal. The mean fluorescence intensity from the FAM channel within these ROIs was measured after background subtraction. Co‐localization analysis was performed to assess the spatial overlap between the TP‐SA and Hoechst signals. The imaging and analysis procedures were identical for all other cell lines investigated.

### 4.14 Induction of Oxidative Stress

HeLa cells were seeded in 12‐well plates at 5 × 10^4^ cells/well. After adherence, the cells were treated with fresh medium containing various concentrations of H_2_O_2_ or MTX for 24 h to induce oxidative stress. Following treatment, the media were removed, and cells were washed with DPBS. OGG1 activity was subsequently measured by incubating the cells with 200 nM TP‐SA for 4 h, followed by processing and analysis via flow cytometry.

### 4.15 Analysis of Clinical BALF Samples

This study was conducted with the approval of the Ethics Committee of Jining NO. 1 People's Hospital (Approval No. 2025‐IIT‐Quick 113, Registration number: MR‐37‐25‐063474). Deidentified, discarded BALF samples were obtained from the Jining NO. 1 People's Hospital Biobank in accordance with guidelines for the collection, transportation, detection, and interpretation of bronchoalveolar lavage fluid in ICU patients. We obtained written informed consent from the patient. Samples were immediately placed on ice and processed by centrifugation at 400× g for 10 min at 4°C to isolate the cellular fraction. The cell pellet was resuspended in RPMI‐1640 medium to a concentration of 1 × 10^6^ cells/mL. The cell suspension was then incubated with 200 nm TP‐SA at 37°C for 4 h. Data were acquired on an Attune NxT Flow Cytometer using the BL1 channel (Ex: 488 nm) to detect FAM fluorescence. Data analysis was performed using FlowJo software (v10.8). OGG1 activity in BALF was quantified by the gMFI of the cell population.

### 4.16 Pharmacological Screening of OGG1 Activators

293T, CT26.WT and HeLa cells were seeded in 12‐well plates. The cells were then treated for 24 h with medium containing 50 µm of the test compounds: TH10785, TH5487, C_8_H_11_N_3_, C_11_H_10_ClN_3_, or C_8_H_13_N_3_S_2_. All compounds were dissolved in DMSO, and the final DMSO concentration in the medium was maintained at ≤0.1% (v/v). Following treatment, cells were washed, and intracellular OGG1 activity was assessed by flow cytometry using the TP‐SA nanoprobe.

### 4.17 Statistical Analysis

For comparisons between two groups with at least three independent biological replicates, a two‑tailed Student's *t*‑test was used. For comparisons involving multiple control groups and a single experimental group, one‐way ANOVA followed by Dunnett's post‐hoc test was employed. For the comparative analysis of OGG1 activity in BALF between patient subgroups, the non‐parametric two‐tailed Mann–Whitney *U*‐test was employed, and *p*‐values were calculated to assess statistical significance. *p*‐values were denoted as follows: ^*^ *p* < 0.05, ^**^ *p* < 0.01, ^***^ *p* < 0.001 and ^****^ *p* < 0.001.

## Author Contributions

All authors were involved in this work. M.Z. and X.S. were responsible for Conceptualization, Validation, and Writing – Original Draft. H.L., B.W., and M.P. were responsible for Methodology and Data Curation. Z.M. and R.K. were responsible for Formal analysis. W.K., Y.Z., and F.Q. were responsible for Recourses, Investigation, Writing – Review & Editing, Project Administration, and Funding Acquisition. All authors approved the final manuscript.

## Conflicts of Interest

The authors declare no conflicts of interest.

## Supporting information




**Supporting File**: advs75847‐sup‐0001‐SuppMat.docx.

## Data Availability

The data that supports the findings of this study are available in the supplementary material of this article.
