# Supplementary material for: An Integrated DNA Nanoprobe for Intranuclear Imaging and in Situ Profiling of OGG1 Activity
Source: Adv Sci (Weinh). 2026 May 27:e75847. Online ahead of print. doi: 10.1002/advs.75847 (PMC13336051; doi:10.1002/advs.75847)
Supplement: Supplementary file 1 — Supporting File: advs75847‐sup‐0001‐SuppMat.docx. [file ADVS-9999-e75847-s001.docx]

**An Integrated DNA Nanoprobe for Intranuclear Imaging and *In Situ* Profiling of OGG1 Activity**

Mingzhu Zhao,^1^ Xuemei Sun,^1^ He Li,^1^ Bangming Wang,^1^ Mengting Pan,^1^ Zhen Ma,^3^ Rong-Mei Kong,^1^ Weiheng Kong,^1,^* Yan Zhao,^1,^* Fengli Qu^2,^*

^1^ Key Laboratory of Life-Organic Analysis of Shandong Province, School of Chemistry and Chemical Engineering, Qufu Normal University, Qufu, Shandong 273165, P.R. China

^2^ School of Molecular Medicine, Hangzhou Institute for Advanced Study, University of Chinese Academy of Sciences, Hangzhou, Zhejiang 310024, P. R. China

^3^ Department of Intensive Care Unit, Jining No. 1 People’s Hospital, Jining, Shandong 272100, China

*Corresponding author.

E-mail address: Weiheng Kong: [kongweiheng@qfnu.edu.cn](mailto:kongweiheng@qfnu.edu.cn); Yan Zhao: [yanzhao2016@hnu.edu.cn](mailto:yanzhao2016@hnu.edu.cn); Fengli Qu: [qufengli@him.cas.cn](mailto:qufengli@him.cas.cn)

**Table of Contents**

Figure S1 S3

Figure S2 S4

Figure S3 S5

Figure S4 S6

Figure S5 S7

Figure S6 S8

Figure S7 S9

Figure S8 S10

Figure S9 S11

Figure S10 S12

Figure S11 S13

Figure S12 S14

Figure S13 S15

Figure S14 S16

Figure S15 S17

Table S1 S18

Table S2 S19

Table S3 S20

Reference S21


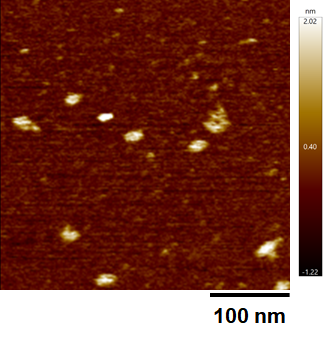


**Figure S1.** The AFM image of the TP-SA nanoprobe.


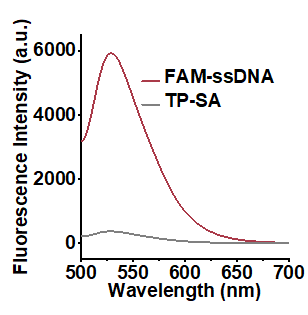


**Figure S2.** Verification of the fluorescence quantitative graph indicating the successful assembly of TP-SA.


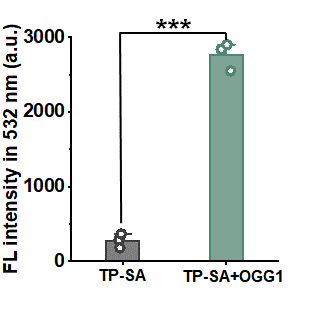


**Figure S3.** Fluorescence quantitative diagram of the response of TP-SA to OGG1.


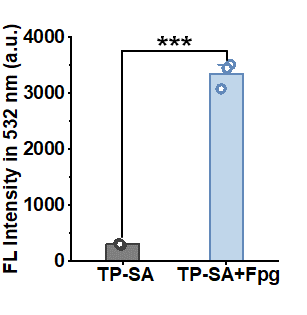


**Figure S4.** Fluorescence quantitative diagram of the response of TP-SA to Fpg.


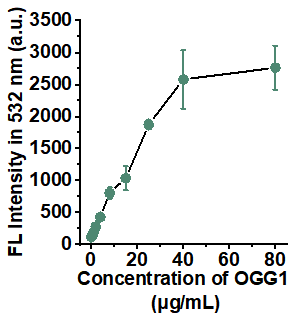


**Figure S5.** Fluorescence dose-response curve of the TP-SA nanoprobe to increasing concentrations of OGG1.


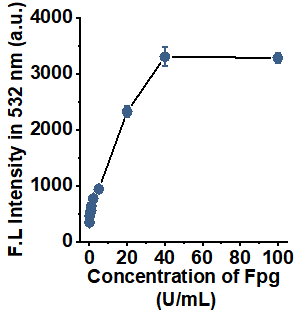


**Figure S6.** Fluorescence dose-response curve of the TP-SA nanoprobe to increasing concentrations of OGG1.


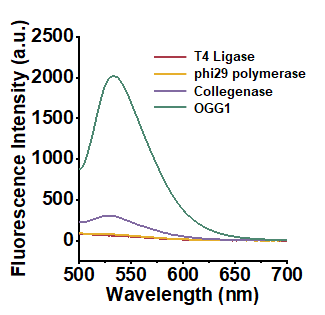


**Figure S7.** The specific fluorescence spectra of TP-SA for OGG1.


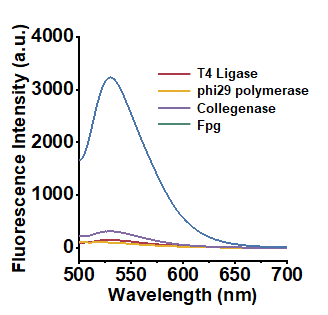


**Figure S8.** The specific fluorescence spectra of TP-SA for Fpg.


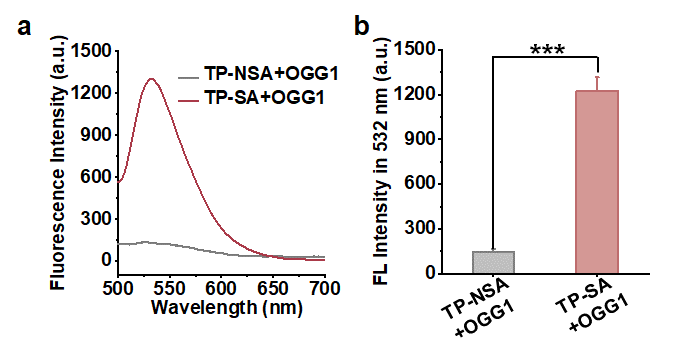


**Figure S9.** The fluorescence spectra (a) and quantitative analysis plot (b) used to verify that the fluorescence response originates from DNA damage-specific repair.


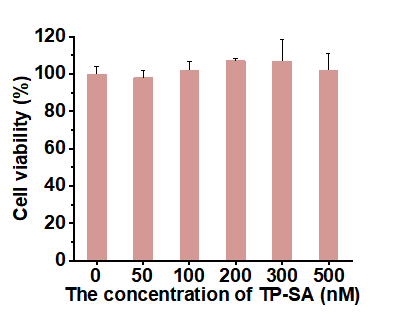


**Figure S10.** The cytotoxicity of TP-SA nanoprobe on HeLa cells.


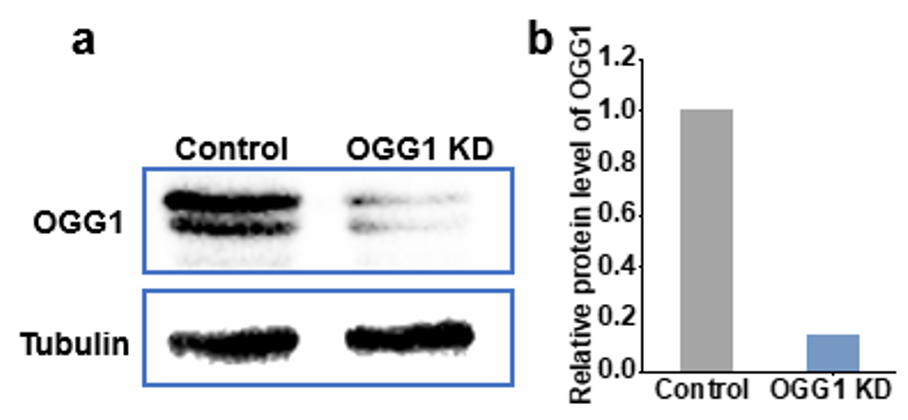


**Figure S11.** (a) The western blot analysis of siRNA-mediated OGG1 expression levels with (b) the corresponding quantitative data.


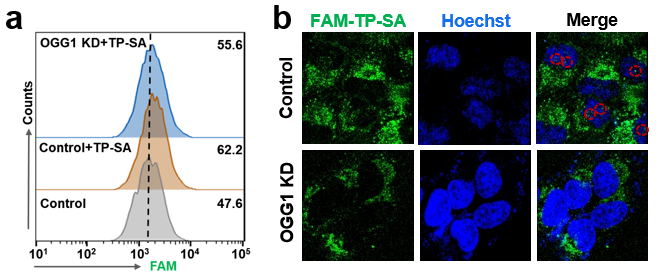


**Figure S12.** Flow cytometry results (a) and confocal images (b) of HeLa cells treated with siRNA and incubated with the TP-SA nanoprobe for 3 h.


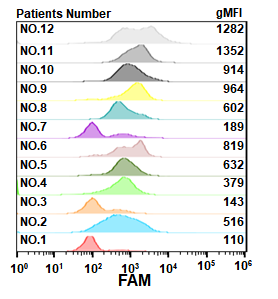


**Figure S13.** The flow chart of TP-SA’s detection of BALF from 12 patients with inflammation.


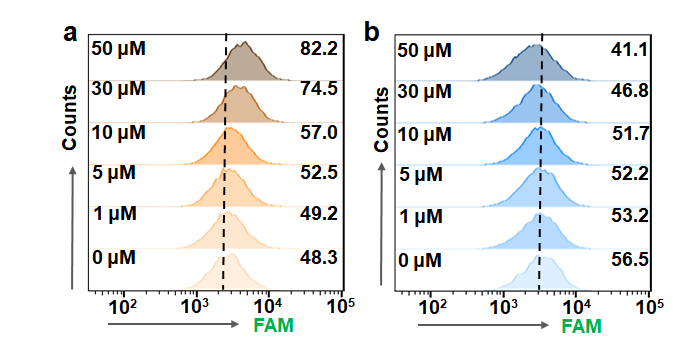


**Figure S14**. Flow cytometry results of HeLa cells treated with different concentrations of TH10785 (a) and TH5487 (b) and incubated with the TP-SA nanoprobe.


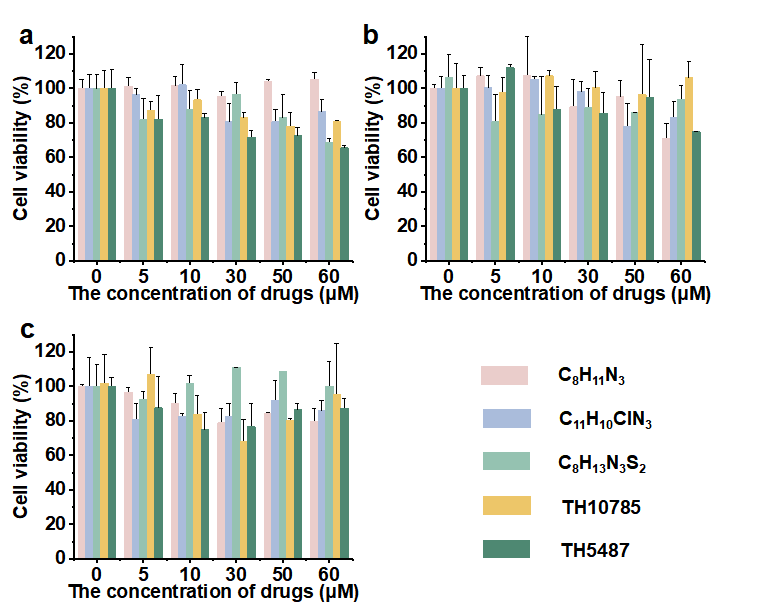


**Figure S15**. The cytotoxicity of different drugs on 293T (a), CT26.WT (b), and HeLa cells (c).

**Table S1**. Sequences of DNA and siRNA oligonucleotides used in this work.

| **Oligonucleotide** | **Sequences (5’-3’)** | **Modification** |
| --- | --- | --- |
| T1 | TCGCTGAGTAttttCCACCACCAAACCACATTTGttttGCATCACTGGGCACCGACACttttCGCACCGCGACTGCGAGGACttttCACAAATCTG |  |
| T2 | CACTGGTGAGttttATCAAGAAGCCGAATTGAAGttttTACTCAGCGACAGATTTGTGttttCGCTCTTCTATACTGGCGGAttttGGTTTGCTGA |  |
| T3 | CCAGTGATGCttttCAACCCACAATCCCAGTGTGttttCTCACCAGTGTCAGCAAACCttttCCATGACGATGCACTACGGGttttGTGTCGGTGC |  |
| S1-A | CAAATGTGGTTTGGTGGTGGtttttACTCAACATCAGGCTAAGACCTGAGT | 3’-FAM |
| S1-B | TCCGCCAGTATAGAAGAGCGtttttACTCAACATCAGGCTAAGACCTGAGT | 3’-FAM |
| S1-C | CACACTGGGATTGTGGGTTGtttttACTCAACATCAGGCTAAGACCTGAGT | 3’-FAM |
| S1-D | GTCCTCGCAGTCGCGGTGCGtttttACTCAACATCAGGCTAAGACCTGAGT | 3’-FAM |
| S1-E | CTTCAATTCGGCTTCTTGATtttttACTCAACATCAGGCTAAGACCTGAGT | 3’-FAM |
| S2 | ACTCAG(8-oxoG)TCTTAGCCTGATGTTGAGT | 5’-BHQ1 |
| AS1411 | GGTGGTGGTGGTTGTGGTGGTGGTGG |  |
| L-AS1411 | CCCGTAGTGCATCGTCATGGttttttttttGGTGGTGGTGGTTGTGGTGGTGGTGG |  |
| N-S (without 8-oxoG) | ACTCAGGTCTTAGCCTGATGTTGAGT | 5’-BHQ1 |
| siOGG1-1 | F: GUUCUGCCUUCUGGACAAUTT  R: AUUGUCCAGAAGGCAGAACTT |  |
| siOGG1-2 | F: GGUGGCUCAGAAAUUCCAATT  R: UUGGAAUUUCUGAGCCACCTT |  |
| siOGG1-3 | F: GCUACGAGAGUCCUCAUAUTT  R: AUAUGAGGACUCUCGUAGCTT |  |

The siOGG1 1-3 sequence is taken from the literature.[1]

**Table S2**. The list of enzyme assays previously reported.

| **Targets** | **Methods** | **Designs** | **LOD (U /mL)** | **Reference** |
| --- | --- | --- | --- | --- |
| Fpg | Fluorescence | Catalytic hairpin assembly | 0.2443 | [2] |
| Fpg | Fluorescence | Recombinase polymerase amplification | 1.12×10^−10^ | [3] |
| UDG | Fluorescence | / | 6.3×10^-6^ | [4] |
| APE1 | Fluorescence | CRISPR/Cas12a | 8.86×10^–4^ | [5] |
| APE1 | Fluorescence | Rolling circle amplification | 0.0005 | [6] |
| This work | Fluorescence | Multiplex signal amplification | 0.07 | / |

**Table S3**. Blood sample reports of clinical patients.

| **Patients** | **WBC (10^9^/L)** | **NEU (10^9^/L)** |
| --- | --- | --- |
| No.1 | 2.46 | 1.94 |
| No.2 | 5.03 | 3.34 |
| No.3 | 5.82 | 3.82 |
| No.4 | 7.65 | 5.8 |
| No.5 | 7.92 | 6.03 |
| No.6 | 10.25 | 6.94 |
| No.7 | 10.92 | 8.97 |
| No.8 | 11.05 | 9.77 |
| No.9 | 13.96 | 13 |
| No.10 | 14.38 | 13.3 |
| No.11 | 14.84 | 11.61 |
| No.12 | 15.02 | 11.93 |

**Reference**

[1] X. Zhou, W. Wang, C. Du, et al.,“OGG1 regulates the level of symmetric dimethylation of histone H4 arginine-3 by interacting with PRMT5,” *Molecular and Cellular Probes* 38 (2018): 19, <https://doi.org/10.1016/j.mcp.2018.01.002>.

[2] Y. Wu, M. Wu, Q. Wang, J. Han, M. Liu,““Repaired and initiated” intramolecular DNA circuit enables the amplified imaging of DNA repair enzyme activity in live cells,” *Sensors and Actuators B: Chemical* 390 (2023): 133992, <https://doi.org/10.1016/j.snb.2023.133992>.

[3] N.-n. Zhao, F.-y. Guo, B.-m. Zhou, M. Liu, C.-y. Zhang,“Construction of a multiple cyclic ligation-promoted exponential recombinase polymerase amplification platform for sensitive and simultaneous monitoring of cancer biomarkers Fpg and FEN1,” *Analytical Chemistry* 97 (2025): 3099, <https://doi.org/10.1021/acs.analchem.4c06344>.

[4] F. Ma, Y.-Z. Liu, M. Liu, J.-G. Qiu, C.-Y. Zhang,“Transcriptionally amplified synthesis of fluorogenic RNA aptamers for label-free DNA glycosylase assay,” *Chemical Communications* 58 (2022): 10229, <https://doi.org/10.1039/d2cc03628b>.

[5] J. Shan, Y. Sheng, L. Luo, et al.,“One-Pot Rlock-Mediated CRISPR/Cas12a-Driven RCA Cycle for Rapid and High-Sensitive APE1 Detection,” *Analytical Chemistry* 97 (2025): 18208, <https://doi.org/10.1021/acs.analchem.5c03234>.

[6] C. Zhao, S. Liu, W. Dang, et al.,“Enzyme-activated biosensor assisted by enzymatic rolling circle amplification for sensitive detection of APE1 and imaging in vivo,” *Analytical Chemistry* 97 (2025): 16690, <https://doi.org/10.1021/acs.analchem.5c03866>.
